# Effective workflow from multimodal MRI data to model-based prediction

**DOI:** 10.1038/s41598-025-04511-5

**Published:** 2025-06-20

**Authors:** Kyesam Jung, Kevin J. Wischnewski, Simon B. Eickhoff, Oleksandr V. Popovych

**Affiliations:** 1 Institute of Neurosciences and Medicine - Brain and Behaviour (INM-7), Research Centre Jülich, Jülich, Germany; 2 Institute of Systems Neuroscience, Medical Faculty, University Hospital Düsseldorf, Heinrich Heine University Düsseldorf, Düsseldorf, Germany; 3 Institute of Mathematics, Faculty of Mathematics and Natural Sciences, Heinrich Heine University Düsseldorf, Düsseldorf, Germany

**Keywords:** Brain MRI, Whole-brain modeling, Parameter optimization, Machine learning, Classification, Prediction, Biophysical models, Computational models, Machine learning

## Abstract

Predicting human behavior from neuroimaging data remains a complex challenge in neuroscience. To address this, we propose a systematic and multi-faceted framework that incorporates a model-based workflow using dynamical brain models. This approach utilizes multi-modal MRI data for brain modeling and applies the optimized modeling outcome to machine learning. We demonstrate the performance of such an approach through several examples such as sex classification and prediction of cognition or personality traits. We in particular show that incorporating the simulated data into machine learning can significantly improve the prediction performance compared to using empirical features alone. These results suggest considering the output of the dynamical brain models as an additional neuroimaging data modality that complements empirical data by capturing brain features that are difficult to measure directly. The discussed model-based workflow can offer a promising avenue for investigating and understanding inter-individual variability in brain-behavior relationships and enhancing prediction performance in neuroimaging research.

**Supplementary Information:**

The online version contains supplementary material available at 10.1038/s41598-025-04511-5.

## Introduction

Since the concept of the human connectome^1^ was proposed almost two decades ago, whole-brain connectivity derived from neuroimaging data has been employed to address questions across various topics including cognitive functions^2^ and brain disorders^3^. An important characteristic of magnetic resonance imaging (MRI) data is their multi-modality that has enabled the researchers to view the brain connectivity from multiple perspectives of structural and functional connections between brain regions^4^. For instance, diffusion-weighted MRI (dwMRI) can be used to investigate the microstructure of white matter as well as to estimate axonal fibers connecting brain regions via tracking streamlines. The latter are interpreted as anatomical connectivity and also referred to as structural connectivity (SC)^5^. On the other hand, resting-state functional MRI (rsfMRI) provides a way to obtain the degree of similarity of activity patterns between brain regions over time, representing functional connectivity (FC)^6^. These two connectivities (SC and FC), constructed in different ways, evidently have different meanings and interpretations and, accordingly, can be utilized in several ways. For example, temporal changes of brain activity will be represented in FC^7,8, ^while anatomical white matter changes in long-term periods can be revealed through SC^9,10^. Furthermore, comparing these connectomes and calculating their similarity led to the notion of the brain structure-function relationship as a possible methodological approach to explore the interdependence between structure and function of the human brain^11^. However, the strength of the structure-function relationship is usually relatively low, might depend on many factors including brain parcellation into separate regions, and its mechanism is still unclear^12,13^.

Integration of model-based approaches into whole-brain connectome research can expand the scope of investigation to understand the brain. The models can, for example, be used to generate simulated FC that together with the fitted model parameters can serve as an additional data modality. This approach provides further attributes that characterize brain dynamics in great detail^14^. In the framework of the whole-brain dynamical modeling, the models were suggested as a possible mediator between brain structure and function, where the empirical SC and FC are used for the model derivation and validation^15^. A natural output of such models is the relationship between simulated and empirical connectomes, which can in particular be used for investigation of the brain structure-function relationship. One of the main advantages of a model-based approach is a great freedom of considering many in silico models, ranges of their parameters and the respective brain activity that may be hidden in a few in vivo measurements^16^. The modeling results may thus contain the information going well beyond that of empirical data and can also validate the biophysical properties of the brain that have been discovered so far or even provide new insights^17^. In addition, with increased power of high-performance computational clusters, a variety of experimental and data-processing conditions can be simulated including modeling of virtual brain interventions in order to identify and test the optimal conditions and parameters, which is hardly possible in vivo^18,19^.

**Fig. 1:**
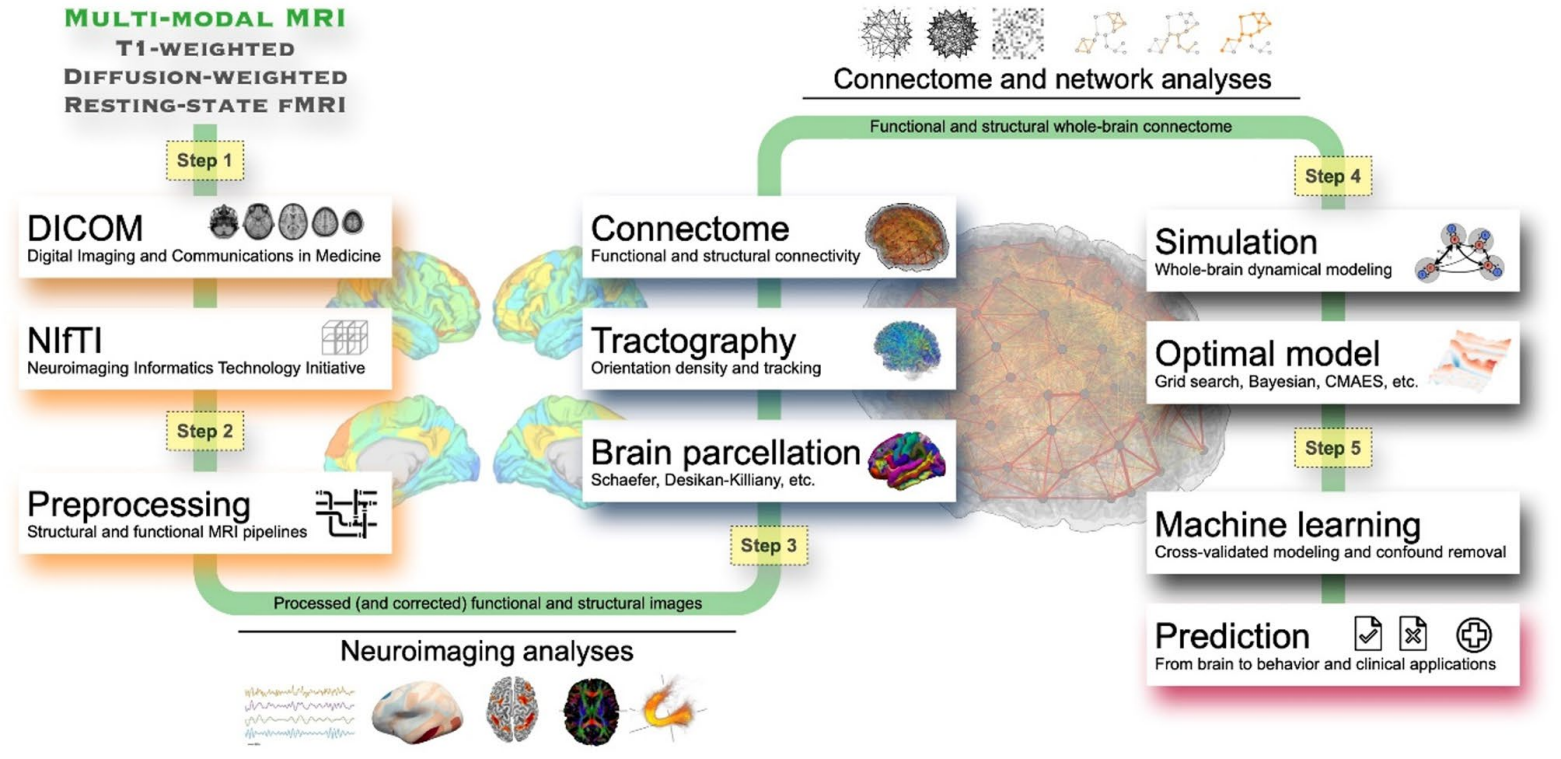
A workflow for model-based prediction research. It can be divided into five steps. The first step is the acquisition of multi-modal MRI data (T1-weighted, diffusion-weighted, and resting-state functional MRI). The second step is preprocessing the acquired MRI data, which can be used for neuroimaging analysis. The third step is to calculate whole-brain tractography and apply a brain parcellation to reconstruct the whole-brain structural and functional connectomes. The fourth step involves whole-brain dynamical modeling including parameter optimization, where the optimal whole-brain model is identified and used to simulate and investigate the brain dynamics in silico. The final step is applying the simulated data for machine-learning analyses.

In this study we suggest a framework that advances the applicability of the model-based approach for neuroimaging research and outline an effective workflow for applying simulated data to machine-learning analysis (Fig. 1). With the suggested framework, we illustrate a few examples of model-based machine learning applied to sex classification and prediction of behavioral scores by employing the data simulated by whole-brain dynamical models. This approach proved beneficial for the performance compared to using solely empirical neuroimaging data. Since the simulated FC and its relationship to empirical FC are among the main outputs of the models, we consider connectome relationships as features for predictions. Purely empirical connectome relationship (empirical SC vs. empirical FC) is used as empirical feature and simulated connectome relationship (empirical FC vs. simulated FC) is used as simulated feature which involves simulated data. We then compare the cases of using empirical features, simulated features, and their combination. Such an enhancement of model applicability might be of relevance, for example, in medical research, where the classification of subjects into patients and healthy individuals might be well assisted by models^20^.

The simulated and empirical connectome relationships exhibit weak similarity between each other with low or even negative correlations across individuals^21^. This indicates that the simulated data showing stronger relationships might contain additional and possibly useful information for the machine-learning prediction analysis if included as features. Along this line, we recently reported that model-based simulated connectomes show higher correlation with clinical scores than that of empirical connectomes, thereby outperforming the latter in this respect^18^.

Including simulated data as an additional data modality in the mentioned studies was motivated by several previous results demonstrating distinct properties of simulated and empirical data in spite of the fact that the models were fitted to the latter. One of the important issues in brain MRI research is the low reliability of findings. This problem has particularly been brought up in the resting-state functional imaging of the whole-brain connectome^22^. However, model-based connectome relationships can offer relatively good reliability and improved subject specificity compared to a fair reliability and low specificity of empirical functional data^23^. Enhanced data reliability might also be important for the prediction analysis^24^. Therefore, applying model-based simulated connectome features, which exhibit distinct patterns along with enhanced reliability and inter-subject variability, to machine learning could lead to consistent results and potentially improved prediction performance as we illustrate in a few examples in this study.

## Methods

In the suggested workflow (Fig. 1), the first step of the model-based approach required multi-modal MRI data, including T1-weighted, dwMRI, and rsfMRI scans. The second step involved processing the MRI data, which included inhomogeneous field/motion corrections, tissue segmentation, cortical rendering, and image registration. In the next step, we applied brain parcellation schemes and computed the whole-brain connectome, including both SC and FC. The fourth step consisted of selecting a dynamical model for the research objectives and optimizing model parameters by fitting simulated data to empirical data. Finally, machine learning was performed using features derived from both the measured and model-based data. We utilized empirical human connectomes, i.e., SC derived from the white-matter fiber tracking and FC calculated by Pearson’s correlation between resting-state Blood Oxygenation Level-Dependent (BOLD) signals of parcellated brain regions. Subsequently, simulated BOLD signals were generated via the considered whole-brain model informed by empirical neuroimaging data and validated by parameter optimization, where the model showed the highest similarity, i.e., Goodness-of-Fit (GoF) between simulated and empirical FCs, and GoF is considered as simulated features which involved simulated data. Then the connectome relationships between empirical and simulated brain connectomes were calculated by Pearson’s correlation between empirical SC (eSC), empirical FC (eFC) and simulated FC (sFC). These connectome relationships were considered as brain features and utilized by machine-learning techniques for prediction of behavioral characteristics of individual subjects, for instance, sex classification or prediction of cognitive scores and five personality traits. The subsections below describe details of each step in the workflow. All methods were performed in accordance with the relevant guidelines and regulations.

### Multi-modal MRI data: step 1

The current study used the Human Connectome Project (HCP) S1200 young adult dataset^25^ including 270 unrelated subjects of 142 females and 128 males with ages in 28.5 ± 3.5 (mean ± standard deviation) years. HCP data were acquired using MRI protocols approved by the Washington University institutional review board (IRB #20124036). Informed consent was obtained from all subjects. Anonymized data are publicly available (https://db.humanconnectome.org). Multi-modal MRI data including T1-weighted MRI (T1w), rsfMRI, and dwMRI were used in the current workflow.

### MRI processing: step 2

A pipeline of MRI processing that consists of structural and functional modules was applied to the multi-modal MRI data, i.e., T1w, rsfMRI, and dwMRI. The pipeline is available via a public repository (https://jugit.fz-juelich.de/inm7/public/vbc-mri-pipeline). The pipeline uses functions in AFNI^26^, ANTs^27^, FreeSurfer^28^, FSL^29^, MRtrix3^30^, and Connectome Workbench^31^. The entire MRI pipeline was aiming at obtaining the whole-brain human connectome. The Schaefer atlas with 100 parcels^7^ and the Harvard-Oxford atlas with 96 parcels^32^ were utilized in this study for brain parcellation in the MNI space. T1w was employed for preprocessing and co-registration between rsfMRI and dwMRI, although it was not directly included in the connectome analysis. However, cortical volumes extracted from T1w were used as a confounding factor in machine learning to classify males and females.

Resting-state BOLD signals were extracted from the rsfMRI processed with FMRIB’s ICA-based X- noiseifier (ICA-FIX) provided by a pipeline of the HCP repository^33^. There were four rsfMRI sessions (1200 volumes, TR = 720 ms) conducted over two different days and consisting of two different phase-encoding directions on each day. In order to obtain the mean regional BOLD signals, the brain was parcellated according to a given brain atlas, and the voxel-wise BOLD signals in every brain region were averaged over all voxels of the region at each time point. A concatenated BOLD signal was then generated by combining all four z-scored BOLD signals from the four rsfMRI sessions.

For the whole-brain tractography (WBT) calculation, response functions were estimated for spherical deconvolution using the constrained deconvolution algorithm^34^. Fiber oriented distributions (FODs) were estimated from the dwMRI using spherical deconvolution^35^, and WBT including 10 million streamlines was created through the fiber tracking by second-order integration over the FOD by a probabilistic algorithm^36^.

### Whole-brain connectome: step 3

For eFC, Pearson’s correlation coefficients between the concatenated regional BOLD signals of each pair of brain regions of the considered brain parcellation were calculated, resulting in the whole-brain resting-state FC. For eSC, the atlases were transformed from the MNI space to the native space of dwMRI. Following the transformation, labeled voxels masked within gray matter were selected for seed and target regions and applied to the WBT. Subsequently, streamlines connecting the seed and target regions were selected for each pair of brain regions, and we ultimately obtained the whole-brain SC matrices including streamline counts and average path lengths of them. With eFC and eSC, we can apply connectome and graph-theoretical network properties for further analyses.

### Mathematical whole-brain model and model fitting: step 4

We simulated a whole-brain dynamical model of $\:N$ coupled phase oscillators^37,38^. Their temporal dynamics can be described by the following set of differential equations:

1 $$\:{\dot{\phi\:}}_{i}\left(t\right)=2\pi\:{f}_{i}+\frac{C}{N}\sum\:_{j=1}^{N}{k}_{ij}\text{sin}\left({\phi\:}_{j}\left(t-{\tau\:}_{ij}\right)-{\phi\:}_{i}\left(t\right)\right)+{\sigma\:\eta\:}_{i},\:\:i=\text{1,2},\cdots\:,\:N.$$

The number of oscillators $\:N$ corresponds to the number of brain regions as defined by a given brain atlas, where $\:{\phi\:}_{i}\left(t\right)$ models the phase of the mean BOLD signal of the corresponding region, and the simulated BOLD was calculated as $\:\text{sin}{\phi\:}_{i}\left(t\right)$. $\:C$ is a global coupling which scales the level of couplings of the whole-brain network. $\:{\eta\:}_{i}$ is an independent noise perturbing oscillator $\:i$, which is sampled from a random uniform distribution from the interval [-1,1]. $\:\sigma\:=0.3$ denotes the noise intensity. The natural frequencies $\:{f}_{i}$ were estimated from the empirical data as frequencies of the maximal spectral peaks (restricted to the frequency range from 0.01 Hz to 0.1 Hz) of the empirical BOLD signals of the corresponding brain regions. $\:{k}_{ij}$ stands for the coupling strength between oscillators $\:i$ and $\:j$, and $\:{\tau\:}_{ij}$ approximates the time delay of the signal propagation between oscillators $\:i$ and $\:j$. They were calculated from the streamline counts and average path-length matrices and determined by the following equations:

2 $$\:{k}_{ij}=\frac{{w}_{ij}}{\langle W\rangle },$$

where $\:{w}_{ij}$ is the number of streamlines between the $\:{i}^{th}$ and $\:{j}^{th}$ parceled region, and $\:\langle W\rangle$ is an average number of streamlines over all connections except self-connections. The delays were calculated as

3 $$\:{\tau\:}_{ij}=\frac{{L}_{ij}}{\langle V\rangle }=\tau\:{L}_{ij},$$

where $\:{L}_{ij}$ is the average path length of the selected streamlines connecting the $\:{i}^{th}$ and $\:{j}^{th}$ region, and $\:\tau\:$ is a global delay, which is a reciprocal of an average speed of signal propagation $\:\langle V\rangle$ through the whole-brain network. The time step of the numerical integration of Eq. 1 by the stochastic Heun method was fixed to 0.04 s, and the simulated signals were generated for 3,500 s after skipping 500 s of the initial transient. The simulated BOLD signals and the corresponding sFC matrices were calculated from the phases down-sampled to TR = 0.72 s, which is the repetition time of the current rsfMRI acquisition.

The considered mathematical model (Eq. 1) has two global parameters: global coupling $\:C$ and global delay $\:\tau\:$. These were optimized within the ranges $\:C\in\:\left[\text{0,1}\right]$ and $\:\tau\:\in\:\left[\text{0,100}\right]$ with the aim to maximize Pearson’s correlation between eFC and sFC. We will refer to this setting with two free parameters as the low-dimensional parameter optimization. Further, we also considered the model fitting in high-dimensional spaces of model parameters, where the noise intensity $\:\sigma\:$ and additional local (regional) parameters of natural frequencies $\:{f}_{i}$  (see Eq. 1) of the brain regions were included in the optimization process. For both scenarios, we applied the Covariance Matrix Adaptation Evolution Strategy (CMAES) for parameter optimization^39,40^. At the parameter optimization by CMAES, the number of particles sampled per generation was chosen as $\:\lambda\:=24$ based on the previous study^41^. To account for a possible result variability of such a parameter optimization, we performed CMAES 30 times for every subject with different initial conditions and selected the optimal model parameters corresponding to the largest GoF for further analyses.

### Machine learning for model-based prediction: step 5

To illustrate the benefits in machine learning via including simulated data into the features, we used the empirical connectome relationship (Pearson’s correlation between eFC and eSC) and the simulated connectome relationship (Pearson’s correlation between eFC and sFC, that is the best GoF of the model to eFC). The empirical and simulated connectome relationships were used for sex classification (*n* = 270) as well as the prediction of cognitive composite scores (*n* = 268, 2 subjects had no cognitive scores) and personality traits (*n* = 269, 1 subject had no data on personality traits) by using machine learning. We also merged the two features (empirical and simulated) and used them for the same machine-learning approach for the classification and prediction analyses. Afterward, we compared the performances with feature conditions of empirical only, simulated only, and merged features.

For the sex classification, we used a nested 5-fold cross-validation (CV) scheme, where every outer CV loop (k = 5) included the embedded 5 inner loops as a nested CV (inner 5-fold CV) for training the prediction model using hyperparameter optimization. The training procedure started with a random splitting of the entire subject sample into 5 equally-sized subgroups while maintaining the ratio of female/male in each subgroup. Subsequently, in every outer loop, one subgroup was selected after another as a testing set, and the other 4 subgroups were united into a training set. In the inner loop with the training set, we performed a confound removal (CR) to remove the effect of brain volumes on the sex classification from the features, i.e., connectome relationships. For this we used the univariate linear regression with the brain volumes (sum of cortical, subcortical and white matter volumes), estimated the parameters of the linear model, and z-scored the obtained residuals across subjects in the training set. Finally, we used a logistic regression with an L2 penalty for the training in the nested CV, and the regularizing parameter was optimized by the Limited memory Broyden-Fletcher-Goldfarb-Shanno algorithm (L-BFGS). After the training in the nested CV, the best model was selected and applied to the testing set to classify the unseen subjects as females or males. Here, the respective CR and z-scoring with parameters obtained for the training set were applied beforehand. Such a CV-CR scheme prevents a data leakage, where no information from the testing set was used during the training^42^. We repeated this prediction process 100 times for different random subject splits into 5 subgroups. Finally, we calculated a balanced accuracy using predicted probability and target variables (female or male).

The CV-CR scheme (5-fold nested CV and CR with brain volumes and ages) was used for predicting the total cognitive function composite score (*CogTotalComp_Unadj*) as general intelligence acquired in the NIH toolbox (https://www.nihtoolbox.org) and also the Five-Factor Model^43^ known as the big five personality traits including openness, conscientiousness, extraversion, agreeableness and neuroticism. The entire group was split into training and testing sets as before while keeping the shape of scores’ distributions for the training and testing sets for an efficient and reliable CV performance^44^. Here, the training and testing sets were created by stratifying the subjects among 7 subgroups balanced within 7 intervals of each target score (cognitive and personality traits) in order to mimic the distribution of the entire cohort. We applied a ridge regression with an L2 penalty for training the prediction model, and the optimal regularizing parameter values were selected among several discrete values of 10^− 6^, 10^− 5^, ..., 10^5^, and 10^6^. The model with the optimal regularizing parameter was selected, which demonstrated the highest Pearson’s correlation coefficient between predicted values and the target scores across subjects in the training set. Consequently, the best model trained through the nested CV on the training set was applied to the testing set to predict the target scores of unseen subjects. We repeated this CV-CR prediction 100 times with each iteration having different stratified subject splits. Finally, we calculated Pearson’s correlation between predicted and measured scores for prediction performance.

For the machine-learning approach, we used Python version 3.11 with modules including Scikit-learn version 1.3.0^45^, NumPy version 1.24.4^46^, and SciPy version 1.11.1^47^.

### Statistical analysis

Effect sizes of the difference between prediction performance of feature conditions were calculated by the Rosenthal formula^48^ which used z-statistics also utilized for calculation of the *p*-values of Wilcoxon rank-sum two-tail test. Bonferroni correction was applied for corrected *p*-values in multiple comparisons. Principal component analysis (PCA) was performed for features, and loadings of each principal component were estimated. All statistical tests and data visualizations were performed in MATLAB (R2024a; MathWorks).

### Code availability

The features for machine learning in this study can be found in the GitHub repository including Python scripts for training prediction models and a MATLAB script to analyze results and generate figures illustrated in this study (https://github.com/kyesam-jung/model-based-prediction).

## Results

By leveraging empirical whole-brain connectomes for the whole-brain dynamical modeling, we successfully generated sFC that can be used alongside the eFC. This allows us to characterize whole-brain dynamics through connectome relationships, highlighting inter-individual variability. Both empirical and simulated connectome relationships can be considered as individual features of whole-brain dynamics and used to classify subjects into different categories or predict their behavioral characteristics using machine-learning approaches. Here, simulated data can complement empirical neuroimaging data or serve as stand-alone features, which can improve the prediction performance. As an example of the proposed framework, we demonstrate that the modeling results can effectively classify subjects by sex (male vs. female) and predict their general intelligence as well as personality traits, showing improved performance compared to using empirical features alone.

### Model-based connectome relationships as leveraged feature information

**Fig. 2:**
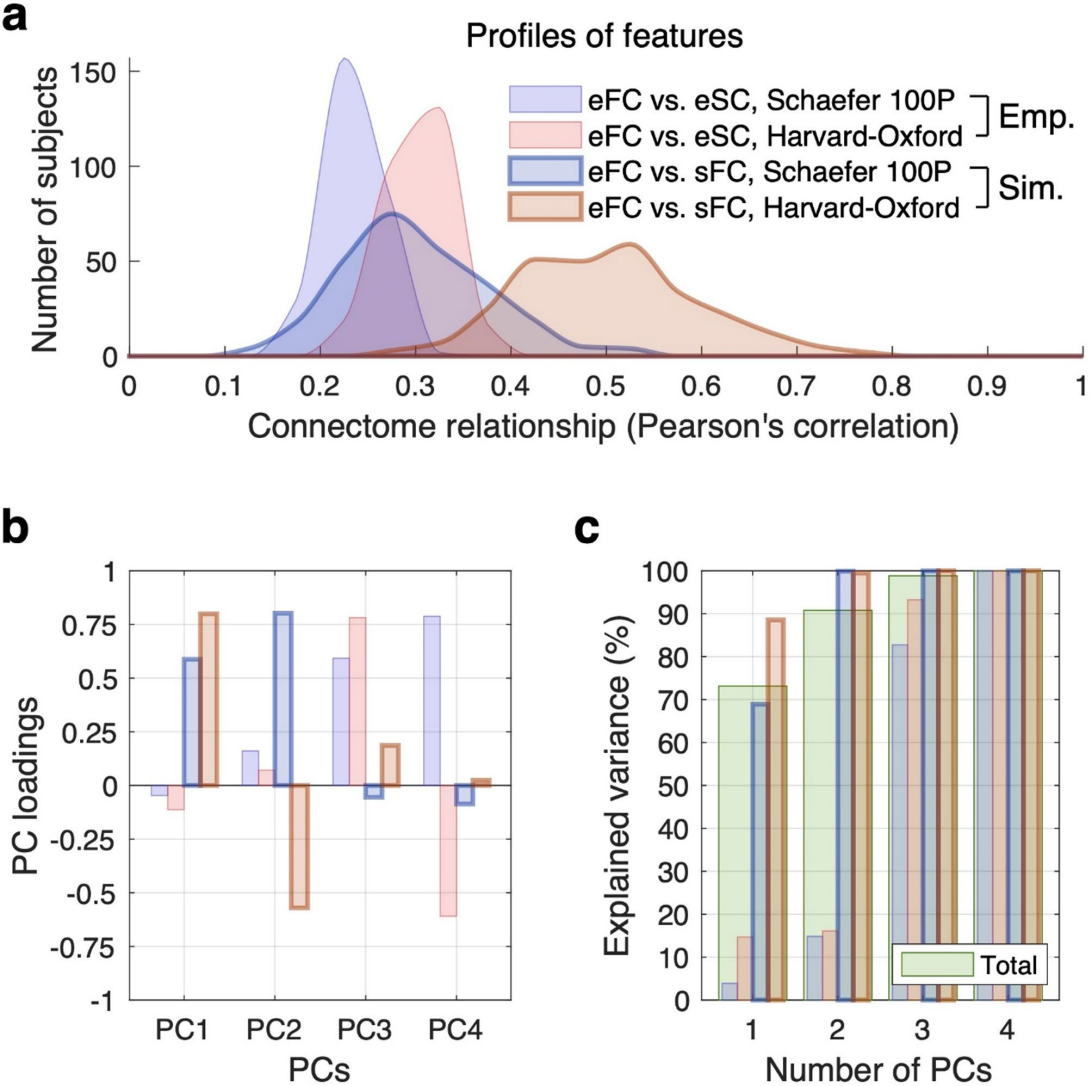
Empirical and simulated features (connectome relationship) for machine learning. (**a**) Feature distributions across individual subjects for two brain parcellations given by the Schaefer atlas (100 regions) and the Harvard-Oxford atlas (96 regions) as indicated in the legend. (**b**,**c**) Principal component analysis (PCA) of the feature variability across 4 feature conditions: empirical, simulated and the two considered brain parcellations. The loadings and the fractions of the explained variance by different principal components are illustrated in plots (**b**) and (**c**), respectively. The color and line schemes are as in plot (**a**). The cumulative explained variance across all conditions is depicted in plot (**c**) by bars in light green color. *eFC* empirical functional connectivity, *eSC* empirical structural connectivity, *sFC* simulated FC, *PC* principal component.

To calculate brain connectomes and their relationships, we utilized two brain parcellation schemes. One is the Schaefer atlas with 100 regions^7, ^where the cortical surface was divided based on functional characteristics of the brain. The other one is the Harvard-Oxford atlas with 96 regions^32, ^where structural brain characteristics were used for cortical parcellation. The connectome relationships as given by the Pearson’s correlation between the respective connectivity matrices were calculated for every subject leading to distributions of their values for a given subject cohort (Fig. 2a). We observed that the two considered parcellation schemes yielded different distributions for the empirical structure-function connectome relationships corr(eFC, eSC) (Fig. 2a, Emp.). In particular, the Harvard-Oxford atlas supported a somewhat stronger structure-function relationship as compared to the Schaefer atlas. Similarly, the simulated connectome relationships corr(eFC, sFC) also produced different ranges of values depending on the parcellation scheme applied (Fig. 2a, Sim.). The considered functional connectome relationship involving the simulated data sFC is important in the brain modeling and frequently used in the literature as a measure of the best fitted model to empirical functional data^15^. We observed that the simulated connectome relationships (eFC vs. sFC) exhibited a much broader spread as compared to the empirical connectome relationships (eFC vs. eSC) including an enhanced inter-individual variability when the simulated data were involved (interquartile ranges, empirical vs. simulated features: 0.033 vs. 0.104 for the Schaefer atlas, and 0.044 vs. 0.136 for the Harvard-Oxford atlas). The effect size of the difference between the atlases was similarly large for both empirical and simulated data (effect size: 1.088 vs. 1.077 for empirical data and simulated data, respectively). Furthermore, the difference between the mean values of each atlas is larger for the simulated data (effect size: 0.679 vs. 1.189 for the Schaefer and Harvard-Oxford atlas, respectively, see Fig. 2a).

The illustrated empirical and simulated connectome relationships can be considered as features for the machine-learning prediction approaches, where the enhanced inter-subject variability of the simulated features (larger spread of the feature distributions) might be a good indication for involving the simulated data in the analyses. To examine the extent of overlap and difference in the feature information under the considered four conditions (2-by-2) shown in the legend in Fig. 2a, we performed PCA using empirical and simulated features of the connectome relationships. Interestingly, we found that the first two principal components (PC1 and PC2), which deliver the largest fraction of the explained variance of all connectome relationships, primarily related to the simulated features (Fig. 2b-c), while the next two components (PC3 and PC4) explained the empirical features. Furthermore, PC1 and PC3 represented common contributing factors in the simulated and empirical connectome relationships, respectively, of the two parcellation schemes (Fig. 2b). In contrast, PC2 and PC4 distinguished the parcellation schemes in the respective simulated and empirical feature conditions (Fig. 2b). The two first PCs with the loading by simulated data cumulatively explained up to 90% of the variance of all features (Fig. 2c). The observed segregation of the empirical and simulated features into different PCs as well as the leading role of the latter features in PCA further support the expectations of a positive contribution of the simulated features to prediction results, which can be used either as stand-alone features or as a complement to empirical ones.

### Classification and prediction performance

**Fig. 3:**
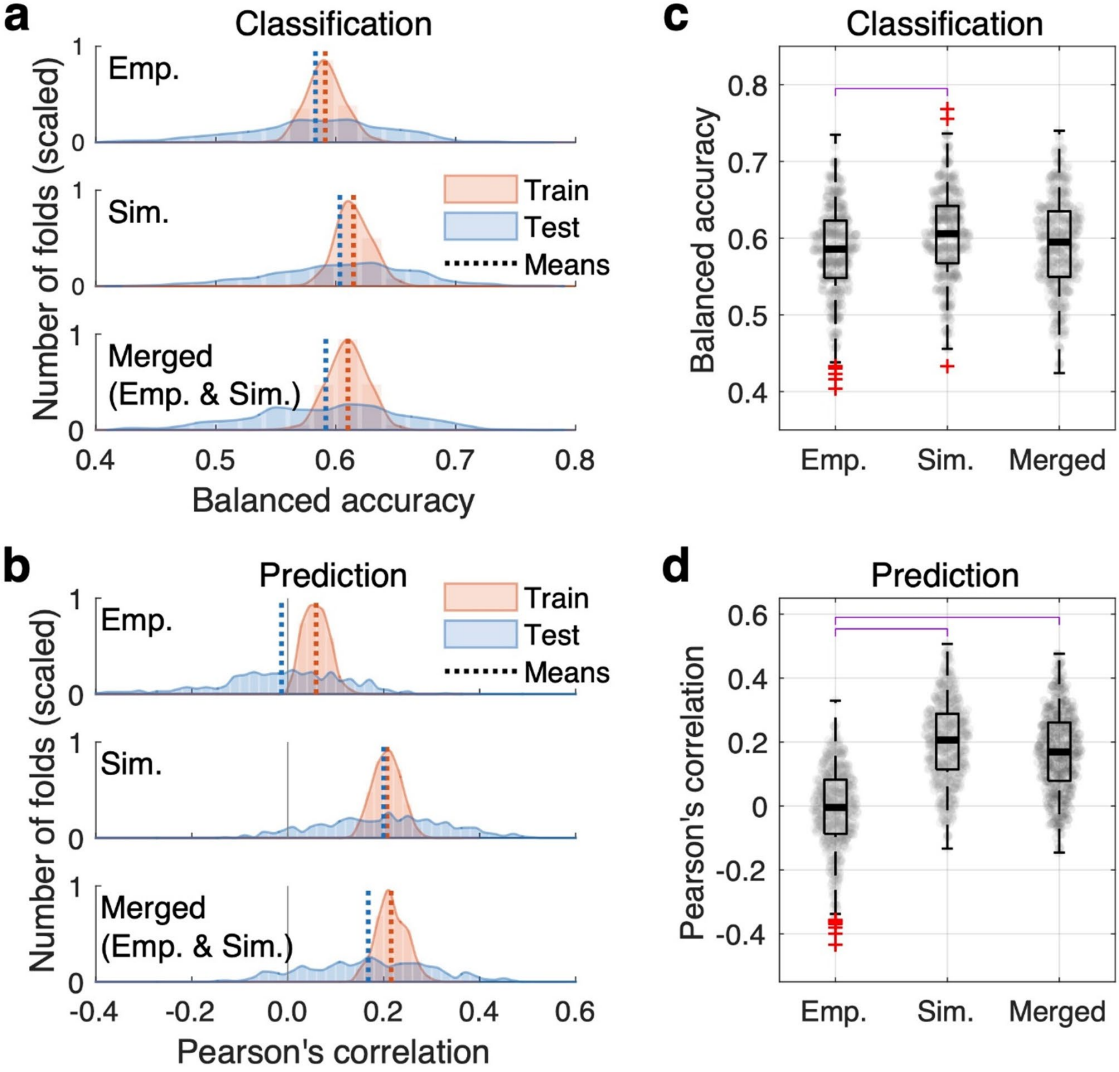
Machine-learning performances in sex classification and prediction of general intelligence. (**a**) Accuracy of sex classification as given by the fraction of correctly classified subjects for the training and testing sets as indicated in the legend. Distributions of balanced accuracy across cross-validation (CV) folds are shown. The mean values of the distributions are indicated by vertical dashed lines. The three plots illustrate the cases of (from top to bottom) the empirical features (Emp.), the simulated features (Sim.), and combination of the empirical and simulated features (Emp. & Sim.). The features of the two parcellations were merged in each condition. (**b**) Prediction of the total cognitive function composite scores (Pearson’s correlation between predicted and empirical scores) for training and testing sets with the same scheme of **a**. (**c**) Comparison of the sex classification and (**d**) prediction performance of the three feature conditions. The magenta bars depict statistically significant differences (with *p* < 0.05 of the Wilcoxon rank-sum two-tail test Bonferroni-corrected for multiple comparisons) in the distribution of prediction between the feature conditions indicated on the horizontal axis.

Since the empirical and simulated connectome relationships exhibit distinct variabilities across individuals (Fig. 2), these two types of connectome relationships might contribute differently to a machine-learning prediction process. To investigate this, we prepared three distinct feature sets: empirical (Emp.), simulated (Sim.), and combined empirical and simulated (Emp. & Sim.) features. Here, the first feature set (Emp.) includes the empirical structure-function relationships (Pearson’s correlation between eSC and eFC), the second feature set (Sim.) includes the relationships between eFC and sFC (GoF values), and the third feature set (Emp. & Sim.) includes both the empirical and simulated features. We then performed two machine-learning analyses using these features to (*i*) classify the individual subjects as females or males and (*ii*) predict a continuous behavioral score as given by the general intelligence based on the total cognitive function composite score^49^. For both cases and under each feature condition, we calculated prediction performance on the training set and after applying the model to the testing set of unseen subjects (Fig. 3a, b). Sex classification on the test subject sets shows that the balanced accuracy was significantly enhanced, when the simulated features were employed in the classification analysis as compared to the case of the empirical features (Bonferroni-corrected *p* < 0.05) (Fig. 3c, compare “Emp.” to " Sim.“). The machine-learning analysis applied to predict the general intelligence also exhibited improved performance with features that contain the simulated data. This was confirmed by statistical tests demonstrating a significant improvement of the prediction performance for the simulated features (Sim.) as well as for a combination of the empirical and simulated features (Emp. & Sim.) compared to the case of the empirical features (Emp.) (Fig. 3d).

### Enhanced performance with high-dimensional parameter optimization

**Fig. 4:**
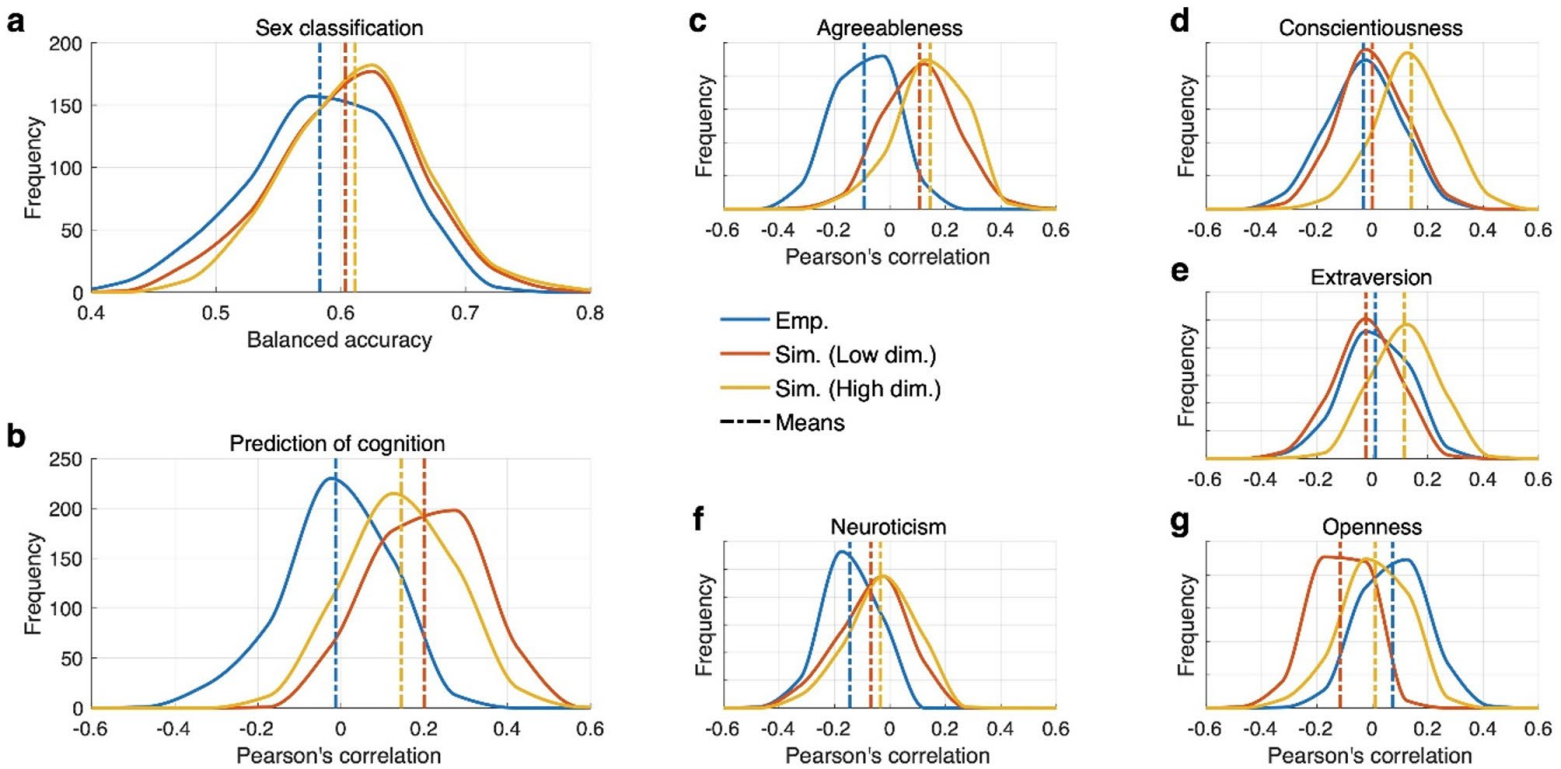
Prediction results with different feature conditions based on empirical features: Emp., simulated features with low-dimensional parameter optimization: Sim. (Low dim.) and high-dimensional parameter optimization: Sim. (High dim.). (**a**) Results of sex classification with five different feature conditions shown in the figure legend in the center. Dashed vertical lines indicate mean values of performance in each feature condition. (**b**–**g**) Results of prediction for cognition (**b** general intelligence) and five personality traits (**c**–**g** agreeableness, conscientiousness, extraversion, neuroticism, and openness).

We also fitted the model to empirical data in high-dimensional parameter spaces, where around 100 model parameters were simultaneously optimized by the CMAES algorithm. In such a way we obtained an increased GoF, where the simulated FCs closely approached the empirical FCs of individual subjects leading to a higher model personalization. For example, the mean GoF = 0.607 and 0.724 for high-dimensional model fitting of the Schaefer and Harvard-Oxford atlases, respectively, may be compared to the respective GoF = 0.299 and 0.501 for the low-dimensional model fitting (Fig. 2a). We then applied the simulated connectome relationships of the high-dimensional model fitting as features to machine learning. Interestingly, the results showed that involving the simulated features obtained through high-dimensional optimization yielded the best outcome in the sex classification (Fig. 4a). Likewise, the low-dimensional optimization condition showed the best prediction of cognition (Fig. 4b). Additionally, in the prediction of personality traits (Fig. 4c–g), the simulated features showed the best results in four out of five traits, except for the openness, where the empirical features demonstrated the best performance (Fig. 4g). These findings indicate that whole-brain dynamical modeling can enhance the performance of machine learning. This is especially evident in predictions of cognitive ability and personality traits, where the empirical features mainly showed correlations near zero, whereas the simulated features demonstrated clearly improved results. To assess the extent to which the results presented in this study align with those predicted by traditional statistical methods, we conducted group comparisons between males and females, as well as linear regression analyses for each feature condition: Emp., Sim. (Low dim.), and Sim. (High dim.). The results demonstrated that the relationships between measured and predicted scores consistently aligned with those obtained through out-of-sample machine learning (Supplementary Figures S1 and S2). Moreover, a permutation test was applied to evaluate the robustness of the current approach (Supplementary Figure S3). We observed that the empirical features can be predictive for sex classification and the openness personality trait only. On the other hand, the performance of the machine learning with simulated features showed a significant and relatively large positive difference from the null distributions except for the openness and neuroticism (Supplementary Figure S3), which is in agreement with Fig. 4.

**Fig. 5:**
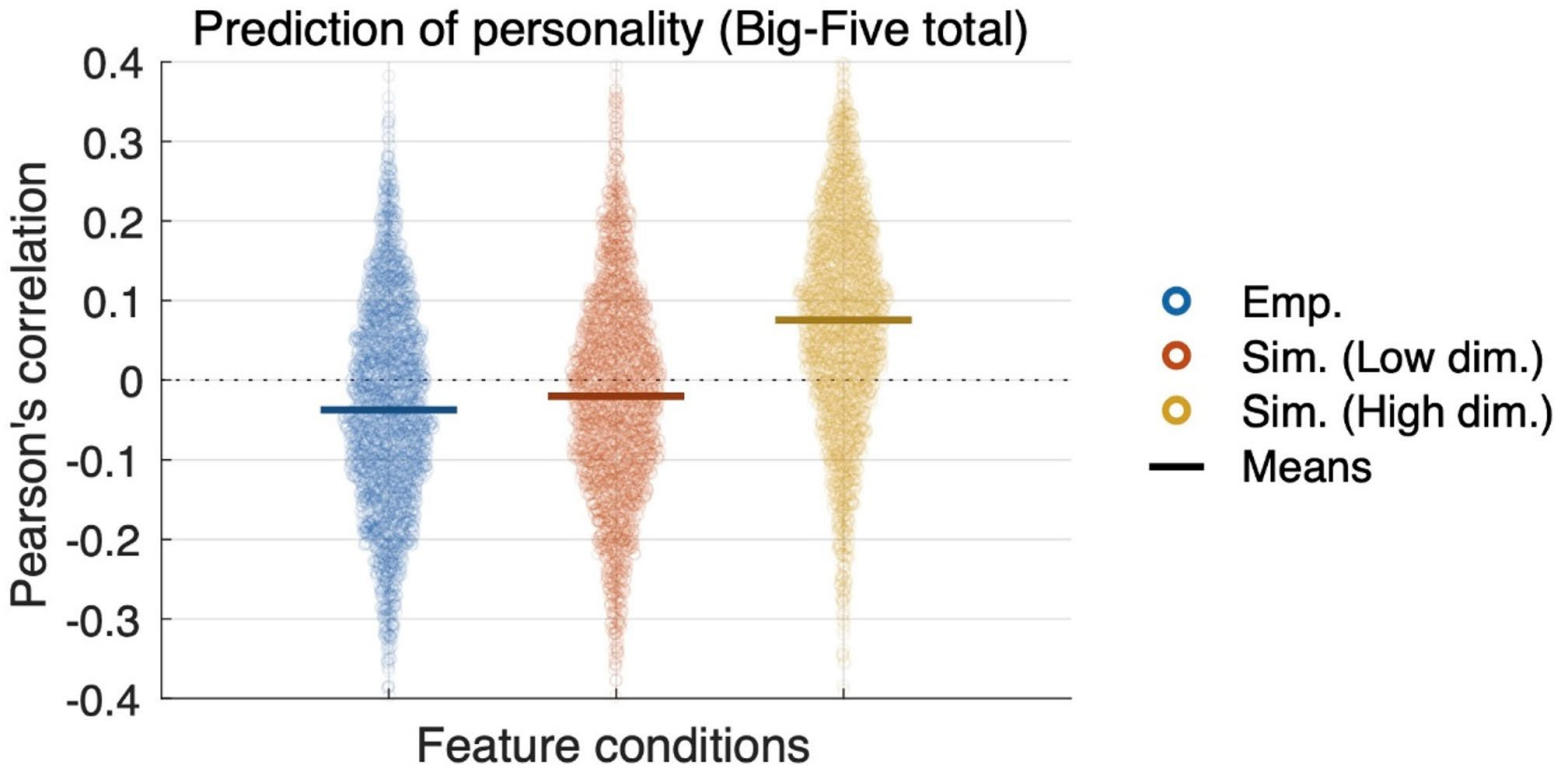
Overall concatenated prediction of personality traits. Each distribution includes Pearson’s correlation coefficients between predicted and measured personality of all five traits in machine learning with 100 iterations and 5 folds cross-validation scheme, leading to 2,500 points.

Finally, when looking at the overall concatenated results of predicting all five personality traits, the simulated features obtained through the high-dimensional parameter optimization showed the highest prediction correlation (Fig. 5), and the difference from the results based on the empirical features was especially large (effect size is 0.836).

The contribution of individual features to prediction results can be examined by analyzing the coefficients of the trained machine-learning models (logistic and ridge regressions, see Methods). This can for example be approached by considering the distributions of the regression coefficients and their contribution to prediction accuracy across all CV loops for all individual empirical and simulated features for the two considered brain atlases (Supplementary Figures S4 and S5). We observe that the feature contributions vary based on the atlas and type of features (empirical or simulated) confirming our conclusions (Fig. 4). In many cases, the good or bad predictability of individual features can clearly be distinguished, see Supplementary Figures S4 and S5 and discussion therein.

**Fig. 6:**
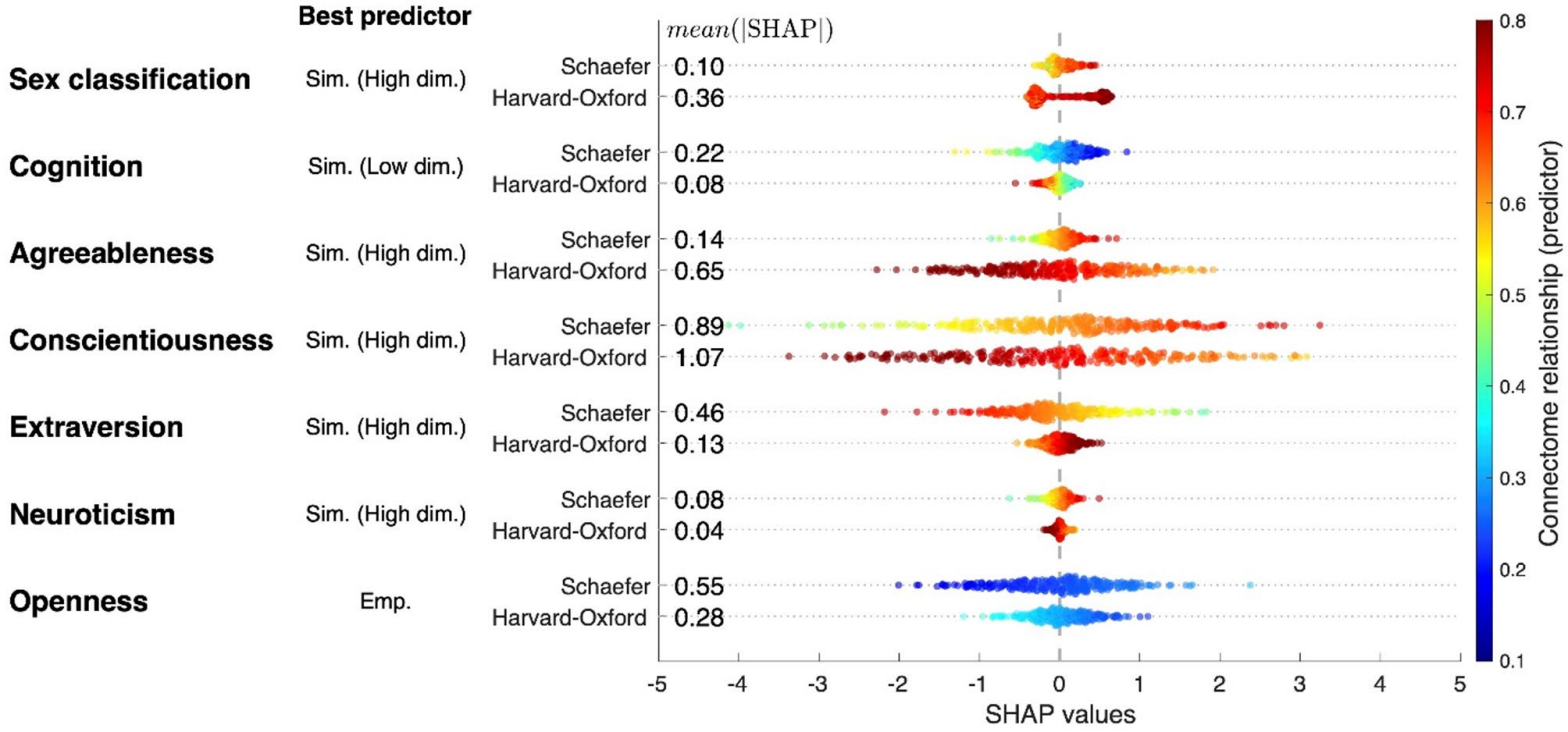
Beeswarm plots of SHAP values for the best predictors of each target. Data points are reflecting the individual subjects, where the SHAP values were averaged across 100 iterations of the nested 5-fold cross-validation for each individual. The vertical axis includes the prediction target (leftmost column), the feature type (middle column) and the brain atlas (rightmost column). Colors of the dots depict feature values of the best predictors with the scaling given on the color bar. The numbers in the plot indicate the mean absolute SHAP values (mean(|SHAP|)) reflecting the SHAP feature importance. The abbreviation ‘Emp.’ is for empirical features, ‘Sim. (Low dim.)’ and ‘Sim. (High dim.)’ are for the simulated features of the low- and high-dimensional parameter optimizations, respectively.

Another approach to understanding feature contributions relates to Shapley additive explanation (SHAP) values^50^ that we calculated for the best predictors of each target (Fig. 6). The latter are empirical features for openness, simulated features of the low-dimensional parameter optimization for cognition and simulated features of the high-dimensional parameter optimization for the other five tasks including sex classification, and prediction of agreeableness, conscientiousness, extraversion, and neuroticism, see Fig. 4 (note the negative mean prediction correlations in Fig. 4f). A comparison between the distributions of SHAP values (Fig. 6) and the distributions of model coefficients (Supplementary Figures S4 and S5) revealed consistent trends. For example, in the case of the agreeableness prediction, the regression coefficients for features derived from the Harvard-Oxford atlas are mostly negative and strongly contributing to prediction (Supplementary Figure S5k). Correspondingly, the distributions of SHAP values under the same condition show the importance of the Harvard-Oxford atlas, where the SHAP values shift from positive to negative as the feature values increase, which is in contrast to the Schaefer atlas (Fig. 6, Agreeableness). As such, all best predictors exhibited a consistent alignment between the distributions of the model regression coefficients and the distributions of SHAP values. We also calculated the mean of absolute SHAP values that reflects the importance of each feature for the prediction results. In summary, the SHAP-based and coefficient-based interpretations can offer complementary insights about the mechanisms of the machine-learning prediction procedure, which can contribute to its better understanding.

## Discussion

In this report, we demonstrated that connectome relationships derived from the whole-brain dynamical modeling can represent individual variability of brain dynamics in a distinct way compared to empirical connectome relationships. We also showed that involving simulated connectomes in the machine-learning prediction analysis can enhance its prediction performance. Furthermore, machine learning using simulated and empirical features in a complementary way exhibited comparable or even improved performance in relation to a separate utilization of these feature configurations. Our results suggest that incorporating model-based features alongside empirical ones can enhance the extent of information extracted from the features provided by neuroimaging data. Building on these findings, it is important to delve into the specific advantages offered by the model-based approach.

The framework for the effective workflow proposed in this study consists of five steps, and the necessary procedures and possible approaches for each step are as follows:

- **Step 1**: For the whole-brain dynamical modeling, three types of MRI data are required: T1w, dwMRI, and resting-state fMRI. For neuroimaging research, raw data, i.e., Digital Imaging and Communications in Medicine (DICOM)^51^ can be converted to a standard format such as Neuroimaging Informatics Technology Initiative (NIfTI)^52^. In addition, the data can be organized according to a consensus data organization called Brain Imaging Data Structure (BIDS)^53^. Nowadays, many published datasets exist, which provide brain MRI necessary for the workflow such as OpenNeuro (https://openneuro.org) or other data collections, e.g., ADNI (https://adni.loni.usc.edu), AOMIC^54^, PPMI^55^ and research projects like 1000BRAINS^56^, HCP^25^, MOUS^57^, PNC^58^, etc.
- **Step 2**: This step of the workflow involves completing the preprocessing of MRI data and signal extractions through a pipeline. In this step, a careful selection of the data processing parameters with high quality control is necessary to check for errors or missing information in the acquired data. Small differences at the early stages can have a significant impact in the final stage of data modeling^20,21,23,59–62^. The pipeline for processing MRI provided in this study (https://jugit.fz-juelich.de/inm7/public/vbc-mri-pipeline) can be configured in various ways depending on the purpose of the study. Alternatively, public pipelines such as fMRIPrep^63^, MRtrix3^30^, QSIPrep^64^, SPM^65^, and FreeSurfer^66^ can be used. The processed data can be utilized to study the functional or structural characteristics of the brain through imaging analysis, as well as for modeling.
- **Step 3**: This step involves parcellating the brain into multiple regions according to a given brain atlas considering various schemes^7,32,67–69^ and calculating functional and structural connectivity of each pair of regions in order to construct the human brain connectome^1^. At this stage, the data necessary for the modeling (step 4) will finally be prepared. A few datasets of BOLD signals, SC and FC calculated for many brain parcellations are available on the EBRAINS (https://www.ebrains.eu) platform ready for analysis and modeling^70–72^. Furthermore, since the structural and functional connectivities between brain regions can be interpreted as underlying structures of the information flow and its processing within the brain networks^73^, studies can be conducted to explore the relationships between network characteristics of SC and FC and behavioral, cognitive and clinical scores^2,3^.
- **Step 4**: A whole-brain dynamical model can be constructed based on the empirical whole-brain connectomes and used to simulate brain dynamics such as electrical neuronal activity and BOLD signals. By varying the model parameters, one can analyze the simulated brain dynamics in comparisons with empirical data using BOLD signals^74, ^FC^15^, dynamic FC that captures evolution of FC over time^75, ^SC^21^, metastability^76^, behavioral or clinical scores^18,20^, etc. This allows us to find optimal model parameters, where the model best replicates empirical brain dynamics and behavior depending on the study objectives. Several software packages are available for the modeling of neuronal brain dynamics, for example, The Virtual Brain^77^, NEST^78^ and DCM^74^ to mention a few. Furthermore, by employing dedicated parameter optimization algorithms^41^, we can obtain fine-tuned models for an improved replication of empirical data. Such a whole-brain dynamical modeling approach provides personalized optimal model parameters after model fitting toward specific target neuroimaging or behavioral scores of individual subjects, thereby showing the strongest relationship between simulated results of optimal models and clinical characteristics^18–20^ or cognitive functions as demonstrated in the present study.
- **Step 5**: This stage involves conducting machine-learning prediction analysis using model-based data obtained from the previous steps. In this step, the cross-validated model-based scheme^20^ extracts effective simulated features derived from personalized optimal models, and their predictive performances are evaluated using machine-learning techniques. This approach allows us to incorporate additional model-based features into the machine-learning process while keeping the established protocols of conventional machine-learning methodologies based on neuroimaging empirical data such as Julearn (https://juaml.github.io/julearn)^79^. The cross-validated model-based machine-learning approach has demonstrated improved prediction performance, as evidenced by medical data^20^ and this study.

In this study, the suggested workflow was applied to sex classification and prediction of behavioral scores for the healthy population of young adults. We report on improved sex classification and prediction of cognition scores by simulated connectome relationship compared to empirical structure-function relationship. Furthermore, the discussed workflow of the model-based prediction led to significant improvement in the prediction of personality traits that were only weakly predicted by empirical structural connectomes^80^ or by the empirical structure-function relationship as shown in this study. In order to better explain the feature contributions to the machine-learning models and prediction results, we calculated the distributions of the regression coefficients of the trained models (Supplementary Figures S4 and S5) and the SHAP values^50^ (Fig. 6), which confirmed our conclusions that the simulated features appeared to be important in most considered prediction cases and also illustrated the role of the considered brain parcellations in the prediction analysis.

The primary objective of this study was to illustrate a model-based prediction approach incorporating personalized whole-brain modeling, where the models were derived from and fitted to neuroimaging data of individual subjects. Within this framework, researchers may utilize either their own models or established ones; however, careful consideration should be given to model-specific dynamics when applying and interpreting the results. For instance, in the present study, high-dimensional parameter optimization may pose a limitation due to potential risk of overfitting if the optimized model parameters obtained for one subject were to be tested on another one. Additionally, the choice of initial parameters for optimization may still have influenced the fitting and prediction results, although we conducted repeated optimizations using different initial parameters to mitigate the variability in the outcomes.

In this study, we propose to consider the simulated data as an additional neuroimaging data modality that captures distinct properties compared to empirical data and can be leveraged for machine learning. In the previous studies, theoretical justifications were demonstrated, where the simulated data clearly exhibited an enhanced inter-individual variability, test-retest reliability and subject specificity compared to empirical data^21,23^. This was in particular demonstrated for a personalized whole-brain dynamical model of coupled phase oscillators, which motivated its consideration in this study and possibly contributed to the improved prediction results. The use of oscillator models is supported by their relevance to brain oscillatory dynamics^81,82^. Biophysical models can also be considered for dynamical modeling potentially leading to translational applications^18–20,83,84^. Beyond this, various studies have used simulated brain dynamics to draw neurobiological interpretations^85,86^. These findings can contribute to a growing body of work using simulated brain dynamics for neurobiological insights and may inform future research on personalized whole-brain modeling and its application to investigation of brain-behavior relationships.

The discussed model-based approach can effectively be used for testing a variety of experimental and data-processing conditions applicable to many topics of brain research^14,19^. This approach has several advantages including enhanced reliability and flexibility as well as cost efficiency as it eliminates the burden to repeatedly acquire whole-brain dynamics from participants under different experimental conditions in the scanner. Additionally, given the diversity of approaches for the whole-brain modeling^87–90^, researchers can select and utilize models that best align with their research objectives, thereby facilitating model-based connectome investigation. For example, we can also apply the proposed model-based prediction approach to other modeling techniques such as behavioral model fitting based on graph-theoretical network properties demonstrating an enhanced correlation with clinical scores when compared to empirical data^18^.

A critical aspect of this modeling process is the selection of data processing pipelines, including brain parcellation schemes and other parameters, which can significantly influence the modeling outcomes^91^. More than 20 brain parcellation schemes have been employed in neuroimaging research, contributing to the diversity of empirical and simulated brain dynamics as well as connectivity^13,21,60^ including the reliability and specificity of the results^23^. Consequently, there is no ground truth or well-justified recommendation for atlas selection for a given neuroimaging analysis, whether based on empirical data or modeling studies. The choice of atlas can also influence machine-learning outcomes, resulting in performance alterations^20,80,92^. It is therefore advisable to involve several brain atlases in contemporary studies in order to confirm and compare the results for other parcellations. In this study, we considered two atlases based on the structural and functional brain properties and providing comparably good reliability and subject specificity for simulated FC^23^. Additionally, merging multiple atlases within the feature space may further enhance performance^20^, which we utilized also in this study. Moreover, variations in neuroimaging processing pipelines can substantially affect research outcomes^59,61,62^, and multiple strategies of model fitting can be applied to optimizing whole-brain models in different ways^18,20,41,93^. The variability of simulated connectomes across subjects can also provide more personalized data across a broader range of perspectives compared to analyses based solely on empirical results^23^.

This workflow has been applied to clinical data as well, where an improved classification performance was reported when simulated features were included in the machine learning^20^. We therefore expect that the suggested model-based approach can be generalized to small clinical cohorts with possibly low-quality neuroimaging data as has already been tested for classification of patients with Parkinson’s disease and correlation with clinical scores^18,20^. The applicability to other datasets still has to be explicitly demonstrated.

By incorporating model-based features alongside empirical data, we can extensively explore brain connectomes and their relationships, offering enhanced performance and other benefits. At the same time, researchers can gain a deeper understanding of the brain dynamics. Given the recent advancements in digital brain research, integrating and expanding brain models^94^, the systematic model-based approach proposed in this report represents a promising method for advancing brain models and their applications. Furthermore, considering modern deep learning methods with enough features extracted from various stages of the proposed workflow including voxel-wise, region-wise and network-wise approaches may enhance the prediction performance, where the models can provide additional features based on the space of model parameters hardly accessible for empirical data. Consequently, this approach underscores the potential for leveraging integrated data to provide comprehensive insights and improved predictive capabilities in neuroimaging research.

## Electronic supplementary material

Below is the link to the electronic supplementary material.

Supplementary Material 1

## Data Availability

The features for machine learning in this study can be found in the GitHub repository including Python scripts for training prediction models and a MATLAB script to analyze results and generate figures illustrated in this study (https://github.com/kyesam-jung/model-based-prediction).
