# Supplementary material for: Effective workflow from multimodal MRI data to model-based prediction
Source: Sci Rep. 2025 Jun 20;15:20126. doi: 10.1038/s41598-025-04511-5 (PMC12181387; doi:10.1038/s41598-025-04511-5)
Supplement: Supplementary file 1 — Supplementary Material 1 [file 41598_2025_4511_MOESM1_ESM.pdf]

Supplementary

Effective workflow from multimodal MRI data to model-based prediction

Kyesam Jung[1,2], Kevin J. Wischnewski[1,2,3], Simon B. Eickhoff[1,2], Oleksandr V. Popovych[1,2*]

[1]Institute of Neurosciences and Medicine - Brain and Behaviour (INM-7), Research Centre Jülich, Jülich, Germany

[2]Institute of Systems Neuroscience, Medical Faculty and University Hospital Düsseldorf, Heinrich Heine University Düsseldorf, Düsseldorf, Germany

[3]Institute of Mathematics, Faculty of Mathematics and Natural Sciences, Heinrich Heine University Düsseldorf, Düsseldorf, Germany

***Corresponding author:** Oleksandr V. Popovych (o.popovych@fz-juelich.de)

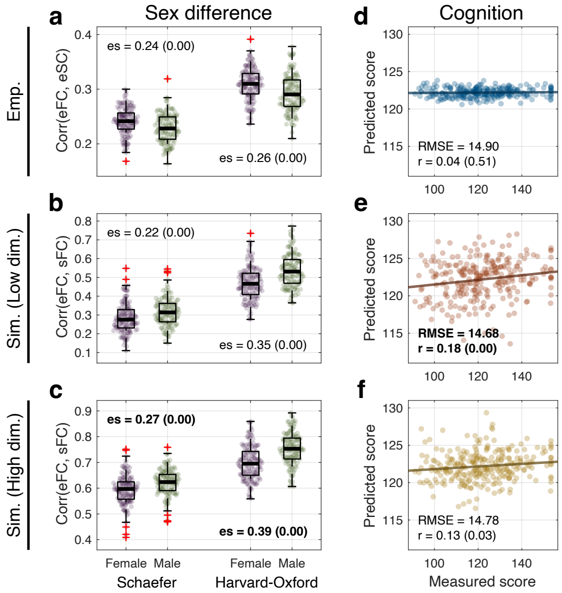

Figure S1. Comparison of the connectome relationships between females and males and linear regression analysis of cognition. (a-c) Distributions of the empirical (Emp.) and simulated (Sim.) connectome relationships for each subject group (females and males) and conditions of the low- and high-dimensional parameter optimizations (Low dim., High dim.) as well as Schaefer and Harvard-Oxford brain atlases indicated on the horizontal axes. Effect size (es) between the groups is shown in the plots with its *p*-value in the parentheses. **(d-f)** Scatter plots of the cognition scores predicted by the multiple linear regression versus the measured scores. The independent variables of the regression were the same empirical and simulated features used for the machine-learning prediction in the main text (Methods and Fig. 4b). Root-Mean-Squared-Error (RMSE) and Pearson's correlation coefficient r with its *p*-value (in parentheses) are indicated in each plot for each feature condition (Emp., Sim. Low-dim, Sim. High dim.), and the best performance is highlighted in boldface.

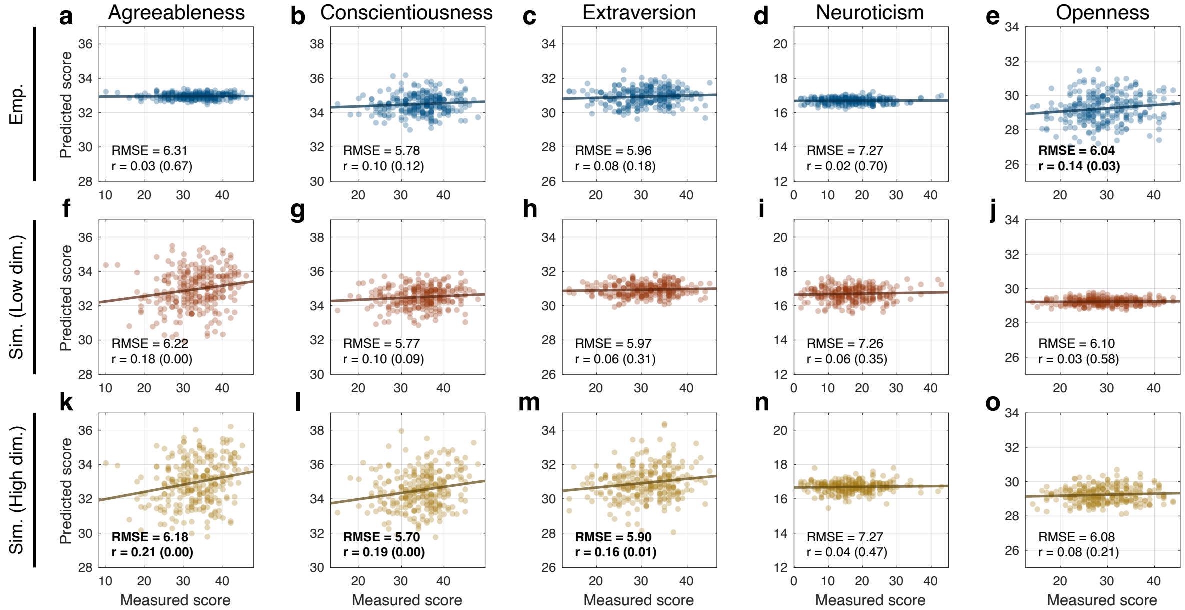

Figure S2. Linear regression analysis on each trait of the personality. (a-o) Scatter plots of predicted scores from linear regression against measured scores. Root-Mean-Squared-Error (RMSE) and Pearson's correlation coefficient 'r' with its *p*-value are shown on each plot for each feature condition, respectively.

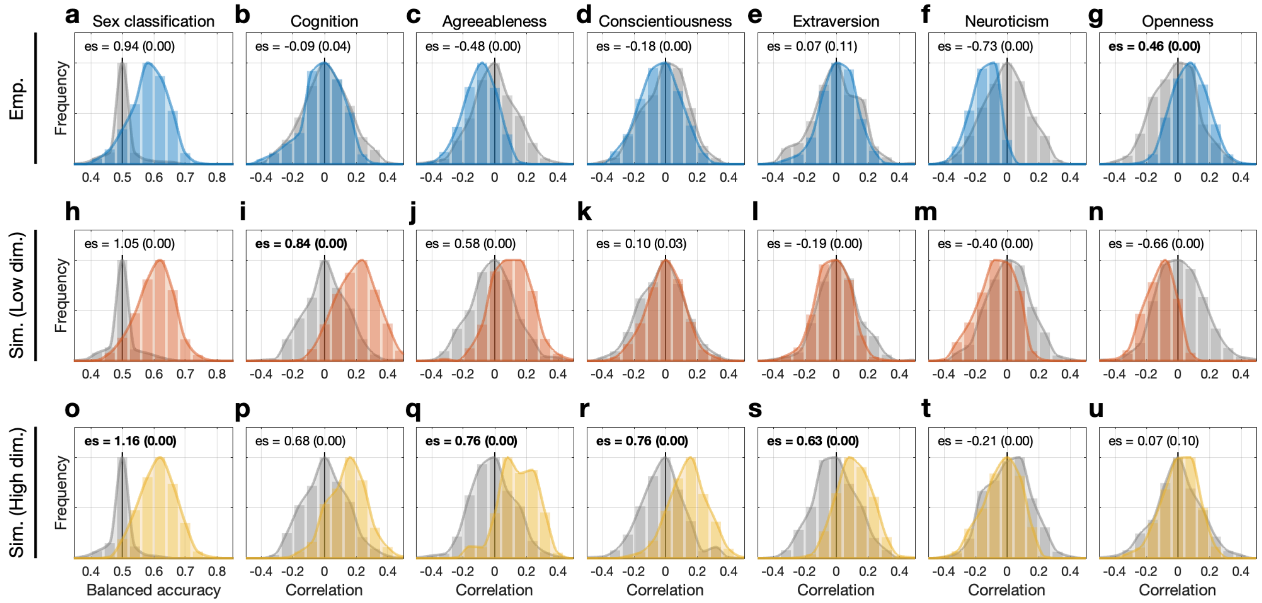

Figure S3. Comparison between permutation tests and prediction results. The null distributions of the permutation tests (gray histograms) were obtained by repeatedly randomly shuffling the target scores among subjects before the machine-learning prediction. The respective permutation tests were compared to the original prediction results (colored histograms) of the balanced accuracy for sex classification (leftmost column) and correlations for cognition and five personality traits for **(a-g)** empirical feature (Emp.), **(h-n)** simulated features (Sim.) for the low-dimensional (Low dim.) parameter optimization and **(o-u)** simulated features for the high-dimensional (High dim.) parameter optimization. Effect size (es) between permutation and prediction data is indicated in the plots together with its *p*-value in the parentheses. Bold font indicates the largest positive and significant effect size for a given target score.

Feature contributions to models based on coefficients of machine-learning models.

To examine the contributions of individual features to prediction, we can investigate coefficients of the regression models applied in this study. In the case of sex classification, the empirical features based on the Schaefer atlas appear to contribute more than that of the Harvard-Oxford atlas. On the other hand, when using simulated features, the Harvard-Oxford atlas seems to be the main contributor to performance (Fig. S4a–c). Similarly, examining the distribution of models' coefficients for prediction of cognition reveals that, for empirical features, both positive and negative values are mixed, while the simulated features consistently show negative values. Moreover, the Schaefer atlas appears to be more prominently utilized in this case (Fig. S4d–f). In a similar manner, the predictive models for personality traits can also be analyzed (Fig. S5). A consistent observation is that models with better performance tend to have coefficient values concentrated either positively or negatively, whereas models with lower performance show coefficients more evenly mixed between positive and negative values. An interesting finding is that, even with the same analytical approach, the manner in which features contribute to prediction can vary depending on the atlas used. In addition, we can elucidate feature contributions to models that show good performance via estimating SHAP values. This metric allows us to interpret the best model how features contribute to the model.

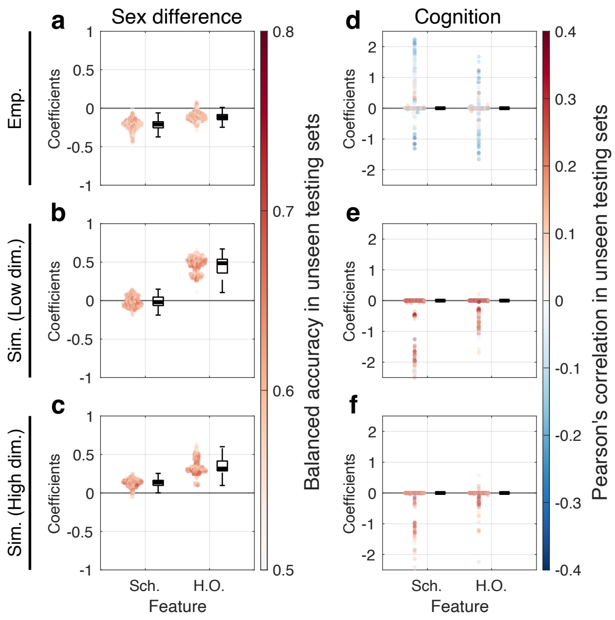

Figure S4. Distributions of the optimized regression coefficients in machine learning for sex classification and cognition prediction. (a-c) Coefficients of the trained models for sex classification. Each distribution reflects separate feature conditions of the two considered atlases indicated on the horizontal axes ('Sch'. for the Schaefer atlas and 'H.O.' for the Harvard-Oxford atlas) for empirical features (Emp.) and simulated features of the low- and high-dimensional parameter optimizations ('Sim. (Low dim.)' and 'Sim. (High dim.)'), respectively. The color gradients of the dots indicate the balanced accuracy obtained for the testing sets. **(d-f)** The same analysis for cognition prediction. The color gradients of the dots here indicate Pearson's correlation between the measured and predicted target scores in testing sets.

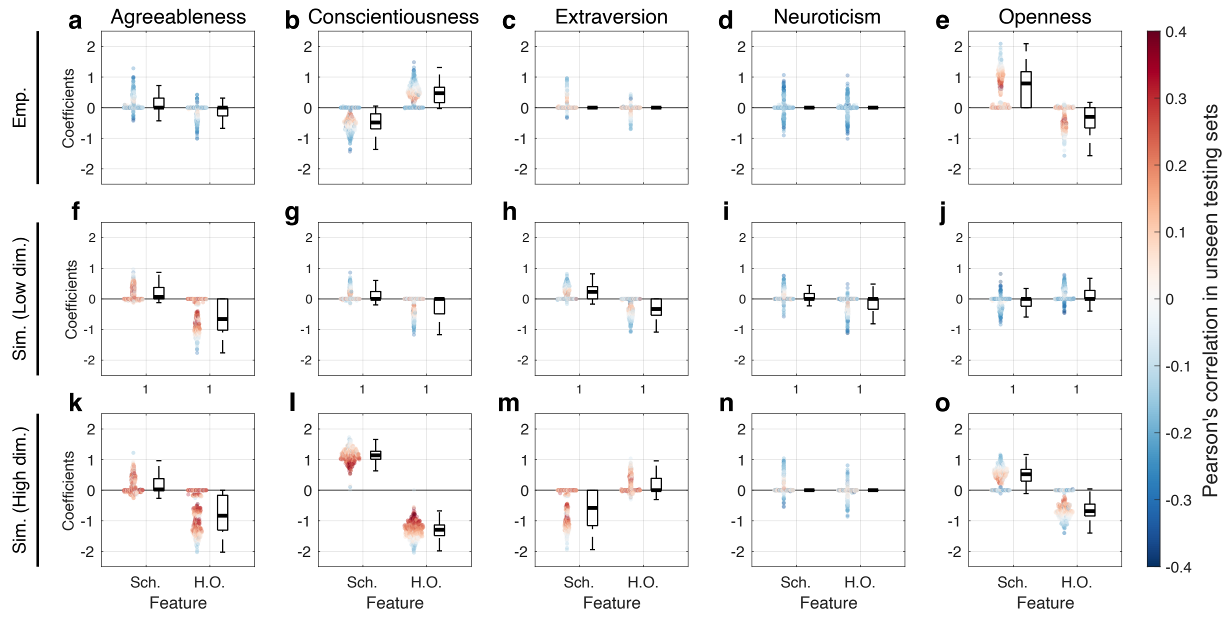

Figure S5. Distributions of optimized regression coefficients in machine learning for prediction of personality traits. (a-e) Coefficients of the trained models using empirical data for the five personality traits. Each distribution shows separate feature conditions of the two atlases. **(f-j)** The same analysis using simulated features of the low-dimensional parameter optimization. **(k-o)** The same analysis using simulated features of the high-dimensional parameter optimization. The color gradients of data indicate Pearson's correlation between measured and predicted scores in testing sets. Other notations are as in Figure S4.
